# Role of Probiotics, Prebiotics, and Synbiotics in the Elderly: Insights Into Their Applications

**DOI:** 10.3389/fmicb.2021.631254

**Published:** 2021-01-28

**Authors:** Elisa C. Ale, Ana G. Binetti

**Affiliations:** Instituto de Lactología Industrial (CONICET-UNL), Facultad de Ingeniería Química (UNL), Santa Fe, Argentina

**Keywords:** microbiota, elderly, probiotic, prebiotic, synbiotic, health

## Abstract

Elderly people are an important part of the global population who suffer from the natural processes of senescence, which lead to changes in the gut microbiota composition. These modifications have a great impact on their quality of life, bringing a general putrefactive and inflammatory status as a consequence. Some of the most frequent conditions related to this status are constipation, undernutrition, neurodegenerative diseases, susceptibility to opportunistic pathogens, and metabolic disbalance, among others. For these reasons, there is an increasing interest in improving their quality of life by non-invasive treatments such as the consumption of probiotics, prebiotics, and synbiotics. The aim of the present mini-review is to describe the benefits of these functional supplements/food according to the most recent clinical and pre-clinical studies published during the last decade. In addition, insights into several aspects we consider relevant to improve the quality of future studies are provided.

## Introduction

Worldwide, elderly people (aged 65 or older) represent 12.4% of the global population (Bedani et al., 2016). According to the European Union, the share of people aged 80 years or above is projected to have a two-and-a-half-fold increase between 2019 and 2100, from 5.8 to 14.6% (Eurostat Statistics Explained, 20201^1^).

This rapid evolution has a significant impact on national public health institutes, social services, and health care systems. Consequently, elderly people are gaining increasing interest since they suffer from chronic health conditions that affect their quality of life, leading to a high demand of health services in general (Bedani et al., 2016). For this reason, new options for preserving their health have been investigated, with being functional food a potential option. In this context, probiotics, prebiotics, and synbiotics are worth studying since the scientific evidence about their beneficial effects on gut microbiota homeostasis is constantly increasing. While probiotics are “live microorganisms that, when administered in adequate amounts, confer a health benefit on the host” (Hill et al., 2014), the term prebiotic is defined as “a substrate that is selectively utilized by host microorganisms conferring a health benefit” (Gibson et al., 2017). From their combination, the term synbiotics arose, which is defined as “a mixture comprising live microorganisms and substrate(s) selectively utilized by host microorganisms that confers a health benefit on the host” (Swanson et al., 2020).

Several phenomena take place during aging, among them, a low-level systemic inflammation during immunosenescence was described by Guigoz et al. (2008). The term “senescence” refers mainly to non-pathological (biological and physiological) processes dependent on age, while the term “aging” refers to physiological and pathological changes (Rowe, 1997; Troen, 2003). In other words, cellular senescence refers to a permanent state of cell cycle arrest that occurs under different stress factors. Therefore, it works as a cellular defense mechanism that prevents cell damage, and it occurs during different physiological (and sometimes pathological) processes, such as tissue remodeling, cancer, injury, and aging (Calcinotto et al., 2019). During immunosenescence, a global reduction in the ability to cope with a wide range of stressors occurs, with a concomitant progressive increase toward a pro-inflammatory status, a process that seems to be mediated by the nuclear factor kappa-light-chain-enhancer of activated B cells (NF-*κ*B factor; Salminen et al., 2008). In addition, inflammatory responses may be caused by the leakage of the intestinal barrier, allowing microbial and/or microbial components to filtrate (Shalim et al., 2019). On the other hand, modifications of the T-cell repertoire have been associated with an increase in morbidity caused by infectious diseases (Vasto et al., 2006), and a low activity of natural killer (NK) cells has been reported as well (Jing et al., 2007).

The gut microbiota of elderly subjects also suffer a gradual shift toward a reduced bacterial diversity: a decline in beneficial microorganisms and an increase of facultative anaerobic bacteria. In general, lower levels of *Firmicutes* (mainly *Clostridium* cluster XIVa and *Faecalibacterium prausnitzii*) and *Actinobacteria* (mainly bifidobacteria), together with increased populations of *Proteobacteria*, have been found when comparing with adults (Salazar et al., 2017). Elderly people may also have reduced dentition and chewing strength, together with a loss of appetite, which can lead to a limited variety of food ingredients that support the limited microbial diversity (O’Toole and Claesson, 2010). These changes are responsible for a decrease in short chain fatty acids (SCFA) production and shift from a predominantly saccharolytic metabolism (normally observed in adults) toward a predominantly putrefactive metabolism (Woodmansey et al., 2004). SCFAs are volatile fatty acids produced by the gut microbiota in the large bowel from food components that are unabsorbed/undigested in the small intestine. They exert beneficial health effects, such as protection against pathogens and shaping the gut environment, apart from presenting anti-inflammatory properties (Ríos-Covián et al., 2016). Furthermore, they have been associated with the upregulation of the anti-inflammatory cytokines *in vitro*, together with the induction of CD4^+^CD25^+^ Treg cells (Asarat et al., 2016).

In this context, considering that the inflammatory status of this group is highly modulated by the gut microbiota (Guigoz et al., 2008) and that external factors such as diet and lifestyle are crucial for this modulation (O’Toole and Claesson, 2010), functional food turns to be an attractive target to study.

In the present review, we intend to revise the latest studies about the application of probiotics, prebiotics, and synbiotics (solely or in different food matrices) on elderly subjects and the effects these strategies have on their health and the quality of life in general. Besides, some guidelines we consider useful for the development of future products aimed at this part of the population are provided.

## Gut Microbiota Composition of the Elderly

According to O’Toole and Jeffery (2015), the composition of microbiota does not suddenly alter at a certain age, but it is a gradual process dependent on several factors, such as gender, location, diet, lifestyle, physical activity, immune system functionality, and the use of medication (O’Toole and Jeffery, 2015; Komanduri et al., 2019). In general, a reduced microbial diversity has been observed, with *Bacteroides* and *Firmicutes* as the most dominant phyla (Claesson et al., 2011; Biagi et al., 2012; Odamaki et al., 2016). Many studies have reported a decline in viable counts of *Bacteroides* with increased age, together with reduced diversity within this genus (Bartosch et al., 2004; Woodmansey et al., 2004; Woodmansey, 2007). This may have a direct impact on digestion since bacteria from this genus are believed to play an important role in the digestion of polysaccharides in the colon (Flint et al., 2012). Furthermore, a decrease in starch and sucrose metabolism, galactose and pyruvate metabolism, and glycolysis/gluconeogenesis has been found using shotgun sequencing, changes that were accompanied by a loss of fibrolytic microorganisms belonging to *Eubacterium*, *Bifidobacterium*, and *Faecalibacterium* genera (Rampelli et al., 2013b). A rise in facultative anaerobes and proteolytic bacteria, such as fusobacteria, propionibacteria, and clostridia, has been reported as well, suggesting a trend toward putrefaction of the large bowel (Woodmansey, 2007). Another characteristic widely observed for this part of the population is a decline in the levels and diversity of bifidobacteria (Woodmansey et al., 2004; Arboleya et al., 2016), possibly leading to a reduced immune responsiveness and an increased susceptibility to gastrointestinal infections (Woodmansey, 2007). In some cases, reduced levels of *Clostridium cluster* XIVa and *Faecalibacterium* were described (O’Toole and Claesson, 2010; Salazar et al., 2013, 2019).

Among the elderly, age seems to be an important factor that determines the microbiota composition, as observed by Salazar et al. (2019). In this study, the levels of *Akkermansia* and *Lactobacillus* for a subgroup of elderly (>80 years old) were significantly higher than those observed in adult (<50 years old) and the younger elderly (50–80 years old) groups, respectively. In this sense, Biagi et al. (2010) found comparable diversity values of the gut microbiota between the elderly and young adults, while centenarians stood out as a separate population, with *Bacteroidetes* and *Firmicutes* still dominating the gut microbiota. However, some changes in the relative proportion of *Firmicutes* subgroups were observed in comparison with the younger adults, with a decrease in the *Clostridium* cluster XIVa, as observed elsewhere (Bartosch et al., 2004; Zwielehner et al., 2009). In addition, the authors described an increase in bacilli, a rearrangement of the *Clostridium cluster* IV (lower levels of *F. prausnitzii* in centenarians than in the younger elderly) and increased *Proteobacteria*. This last group contains many “pathobionts” bacteria, which, under some circumstances (e.g., inflammation), might induce pathology (Biagi et al., 2010). Some members of this group are *Helicobacter hepaticus*, segmented filamentous bacteria, *Escherichia coli*, and *Enterococcus faecalis* (Jochum and Stecher, 2020). Regarding SCFA production, several butyrate producers were found in lower amounts in centenarians than in other age groups, indicating a general decrease in SCFA levels with age (Salazar et al., 2013, 2019).

## Probiotics, Prebiotics, and Synbiotics: Potential Applications

Bifidobacteria and lactobacilli have been widely considered health-promoting constituents of the microbiota (Bedani et al., 2016). Different strains of these genera were lately used as probiotics and proved to have many health benefits within the elderly, such as microbiota modulation, improvement of bowel movements, control of opportunistic bacteria, positive effects on mental conditions, stimulation of the innate immune system, increased vitamin intake, among other effects detailed in Supplementary Table S1 according to clinical and pre-clinical trials. Although the impact of prebiotics and synbiotics has been studied to a lesser extent, there are some recent clinical studies indicating positive health benefits as well. Several attempts to isolate probiotic strains from elderly people have been reported; for example, Silvi et al. (2003) isolated *Limosilactobacillus fermentum* and *Bifidobacterium longum* strains from elderly people (aged 65–87 years, Italy) as part of an EU-funded project, whose final objective was the future application of the isolated strains to design appropriate functional foods for the elderly. Similarly, Park et al. (2015) isolated *L. fermentum* as the most frequent species in fecal samples from longevity (>80 years) populations in Korea, highlighting the potential relevance of this particular species for the formulation of probiotic food or supplements for seniors.

### Effects Demonstrated on Elderly Subjects by Clinical Trials

Most of the latest research carried out in this field implied clinical trials addressed to healthy elderly people (Supplementary Table S1). In general, the application of commercial probiotics (one strain or a cocktail) was the most chosen strategy among them. The effects observed indicate that the consumption of probiotics may positively impact the gut microbiota by increasing the levels of bifidobacteria or modifying subpopulations of lactobacilli (Nagata et al., 2011; Akatsu et al., 2013; Ostan et al., 2015). Furthermore, probiotics were associated with the ability to promote interactions between key constituents of the microbiota and the host epithelium (Eloe-Fadrosh et al., 2015), enhance the immunity response (Finamore et al., 2019; Yamamoto et al., 2019; Wang et al., 2020) and improve bowel movements (Nagata et al., 2015; Inoue et al., 2018; Aoyagi et al., 2019). Other health benefits were related to their ability to revert age-related increase of opportunistic pathogens, such as *Clostridium difficile*, involved in antibiotic-associated diarrhea that impact on nutrition and inflammatory status, exerting an important role in pathophysiological processes. In the elderly, *C. difficile*-associated diarrhea was linked with a reduction on the number of bifidobacteria (Hopkins and MacFarlane, 2002); for this reason, therapies based on the use of probiotics to correct the microbiota imbalance would be promising (Rampelli et al., 2013a). In this direction, Nagamine et al. (2019) reported that probiotics could reduce *Clostridium difficile* infection (CDI) among elderly patients who underwent proximal femoral fracture surgery, but at this moment, there is not enough information about their mechanisms of action, and the current guidelines do not recommend their administration (McDonald et al., 2018). Notwithstanding the promising results, other studies reported controversial ones, most of them having no significant results (Mallina et al., 2018; Sofian et al., 2019).

#### Extra-Intestinal Effects

When considering the extra-intestinal positive effects, probiotics were able to enhance the oral health of elderly by the control of *Candida* and hyposalivation, common problems among this group (Ishikawa et al., 2014; Kraft-Bodi et al., 2015; Lee et al., 2019). Other reports have also associated probiotic consumption with positive effects on respiratory tract infections by reducing the duration (Guillemard et al., 2010; Fujita et al., 2013) or accelerating the healing process in patients with acute distal radius fracture (Lei et al., 2016). Although probiotics showed potential effects on bone metabolism in different mouse models (Roberts et al., 2019, 2020), further clinical trials are required to assess this effect on the elderly.

Recently, brain-gut axis has received special attention, since the application of some probiotics on elderly subjects improved mental conditions, such as anxiety and depression, when combined with a 12-week resistance-training program which consisted of classes comprising a warm-up session, resistance training, and a cool-down session (Inoue et al., 2018). Probiotics also promoted mental flexibility and alleviated stress (Kim et al., 2020). Particularly, they were recommended for the treatment of different health conditions as the silent systematic inflammation and neuroinflammation that are frequently observed in the early stage of Alzheimer’s disease (Leblhuber et al., 2018). These authors reported positive results that indicate a probiotic supplement influenced by gut bacteria composition (increased levels *F. prausnitzii*), tryptophan metabolism and immune response. In this case, in spite of its limitations (a small sample size and the absence of placebo control), it was suggested that the increase of *F. praunitzii* could mitigate the cerebral accumulation of β-amyloid and lipopolysaccharides, which are overproduced during the pathogenesis of Alzheimer’s disease.

Other effects that probiotics exert on the elderly were associated with augmented levels of vitamins in the blood. For example, Valentini et al. (2015) reported increased concentration of vitamin B12 and folate in serum, accompanied by a reduction in plasma homocysteine in subjects that received a commercial probiotic supplementation. The lack of one of these vitamins may cause megaloblastic anemia and a series of neurological and mental symptoms in the elderly, as both vitamins play a crucial role in the cognitive function (Lokk, 2003). These increases were positively correlated with the change in fecal bifidobacterial concentrations for the subjects with low-grade inflammation. The authors suggested that, since the decrease in homocysteine levels was clinically relevant, the probiotic could provide protective effects against some aging-associated conditions (such as cardiovascular or neurological diseases). However, several limitations can be mentioned: It was an open label study without a placebo group, and it used biological instead of clinical endpoints.

On the other hand, the use of synbiotics (combination of probiotics and prebiotics) has also demonstrated similar beneficial effects on the gut microbiota (Supplementary Table S1). For example, synbiotics proved to increase the number of bifidobacteria and lactobacilli, improve the stool frequency and mucosal integrity, increase butyrate production, diminish pro-inflammatory response, and enhance lipid metabolism (Björklund et al., 2012; Granata et al., 2013; Macfarlane et al., 2013). In addition, synbiotics significantly decreased metabolic syndrome prevalence, several cardiovascular risk factors and markers of insulin resistance in elderly patients (Cicero et al., 2020). Regarding prebiotics, some clinical trials demonstrated that they have positive effects on the gut microbiota composition and immune responses as well (Walton et al., 2012; Alfa et al., 2017).

**Figure 1:**
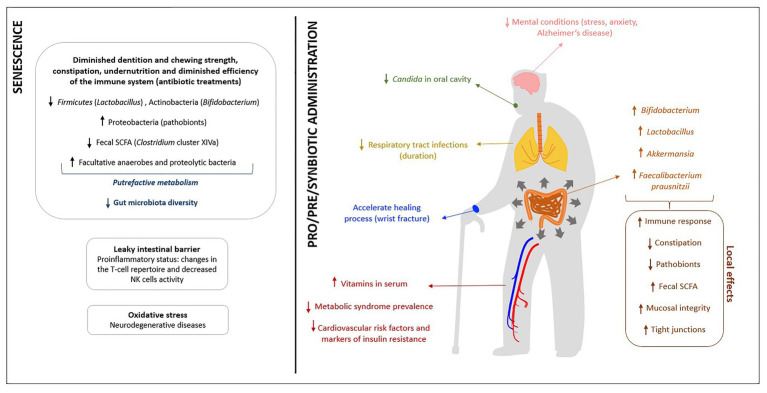
Summary of the main health effects attributed to probiotics, prebiotics and synbiotics in the elderly.

To sum up, Figure 1 shows, as a graphical representation, the physiological, nutritional and immune targets of intervention generally identified for elderly people and the possible effects of these functional supplements on this population.

### Effects on Different Mouse Models

Recently, there have been several studies about the health benefits that probiotics exert on the elderly using different pre-clinical (*in vivo*) models, as summarized in Supplementary Table S1. In general, the most selected model for studying the advanced age is C57BL/6J mice, aged 18 months or more (Flurkey et al., 2007). With this murine model, along with other *in vitro* assays, Ahmadi et al. (2020) demonstrated that a probiotic cocktail prevented mice into undergoing a high-fat diet from microbiota dysbiosis, leaky gut, inflammation, and metabolic and physical dysfunctions. In this direction, Jeong et al. (2016) obtained similar results for a probiotic strain of the species *Levilactobacillus brevis* since the treatment was effective in modulating the gut microbiota, inhibited the expression of inflammatory markers, enhanced colonic tight junctions, and ameliorated colitis and memory impairment. Similarly, Vemuri et al. (2019) demonstrated that a probiotic strain of *Lactobacillus acidophilus* increased the abundances of beneficial bacteria, such as *Akkermansia* spp. and *Lactobacillus* spp., and enhanced the levels of butyrate while downregulating the production of inflammatory cytokines. An interesting result observed by Lee et al. (2016) indicated a probiotic strain provided by female C57BL/6J mice with healthy skin, active folliculogenesis, and hair growth, together with immunomodulation. Probiotics supplementation has also been associated with a positive impact on oxidative stress and inflammation in peripheral tissues in this strain of mice (Ni et al., 2019).

As shown in Supplementary Table S1, there are other murine models that were successfully applied, one of them consisting of using D-galactose to induce premature senescence on Sprague Dawley rats. The results suggest probiotics ameliorated aging-induced metabolic diseases, pathogens growth, microbiota dysbiosis, oxidative stress, inflammation, and alteration of gut metabolites (Hor et al., 2019a,b; Lew et al., 2020). In other works, the BALB/c strain was used, with or without D-galactose injection. Improvement of immunological markers (Molina et al., 2016), modulation of microbiota and protective effects on oxidative stress induced by D-galactose (Zhang et al., 2017) were reported with this model.

Finally, there are some other murine models used for specific studies. This is the case of Yang et al. (2020), who used SAMP8 mice to study the potential of probiotics to treat deficits of the microbiota-gut-brain axis and cognitive function in aging. On the other hand, transgenic B6 mice were used to analyze the effects probiotics have on Alzheimer’s disease, showing promising results on the glucose metabolism (improved glucose uptake) and on the disease progression (Bonfili et al., 2020).

## Parameters to Consider for Future Studies

From the analysis of the information provided in the present mini-review, guidelines to address future studies regarding the role of probiotics, prebiotics and synbiotics in aging could be proposed. Without focusing on specific age groups, the minimum criteria that apply exclusively to probiotic strains for their use in foods and dietary supplements were recently revised (Binda et al., 2020). Similar principles could be considered for the administration of probiotics, prebiotics, or synbiotics to elderly people with special focus on their particular needs.

Special attention should be payed when designing the experiences to ensure the reliability of the clinical trial itself and the correct publication of the results, which should be based on recognized guidelines, as the Good Clinical Practice guidelines of the International Council for Harmonization, ICH-GCP,^2^ and The Consolidated Standards of Reporting Trials.^3^ Some of the factors to consider are (i) the choice of adequate controls, (ii) blinding, (iii) design, and (iv) the selection of the elderly population sample (health or disease status, male or female). In order to diminish the variability among studies, researchers should be aware of the effect different ages could cause, since significant differences between young elderly people (65–80 years old), those aged >80 years and centenarians, have been previously reported (Biagi et al., 2010; Salazar et al., 2019). For this reason, homogenous groups are recommended to avoid skewing the results obtained. On balance, nutritional strategies for the elderly should be addressed from a holistic point of view considering their special nutritional needs, the high susceptibility to disease, and the frequent medicine (mainly antibiotics) intake, as a whole (Salazar et al., 2017).

The location where the research takes place has an important influence (elderly living in their homes, in a hospitalized environment, developed or developing countries, etc), since parameters such as diet and lifestyle are of great relevance (O’Toole and Jeffery, 2015; Salazar et al., 2017). In addition, the matrix in which probiotics, prebiotics, or synbiotics are delivered should be chosen carefully because it may have an impact on the overall results. Diminished dentition, chewing strength, and constipation are essential factors to consider when choosing the matrix. The dose, administration schedule, and duration of intervention must be contemplated as well, as too long treatments might be difficult to be followed in practice, this affecting the health effects expected. Therefore, functional food for seniors, containing probiotics, prebiotics, or synbiotics, should cover all these aspects and should be available in the market as more personalized treatments than products for the public in general.

## Conclusion

The present work suggests that modifying the gut microbiota of the elderly population by the intake of functional food/supplements as probiotics, prebiotics, or synbiotics may be an effective and non-invasive strategy to counteract the natural consequences of aging, in most cases affected by the extended use of antibiotics, providing a better quality of life. At the same time, these functional products may be suitable, affordable, and economical to most elderly people. However, several concerns should be considered when future studies are addressed, to obtain not only reliable results but also treatments feasible to be applied and practical to be followed.

## Author Contributions

EA and AB conceptualized and wrote the manuscript. All authors contributed to the article and approved the submitted version.

### Conflict of Interest

The authors declare that the research was conducted in the absence of any commercial or financial relationships that could be construed as a potential conflict of interest.

## Footnotes

^1^https://ec.europa.eu/eurostat/statistics-explained/index.php?title=Population_structure_and_ageing&oldid=502987

^2^https://ichgcp.net

^3^http://www.consort-statement.org/consort-2010
